# Maternal gatekeepers: How maternal antibody Fc characteristics influence passive transfer and infant protection

**DOI:** 10.1371/journal.ppat.1008303

**Published:** 2020-03-26

**Authors:** Stephanie N. Langel, Claire E. Otero, David R. Martinez, Sallie R. Permar

**Affiliations:** 1 Duke Human Vaccine Institute, Duke University Medical Center, Durham, North Carolina, United States of America; 2 Department of Pediatrics, Duke University Medical Center, Durham, North Carolina, United States of America; 3 Department of Epidemiology, University of North Carolina at Chapel Hill School of Public Health, Chapel Hill, North Carolina, United States of America; Mount Sinai School of Medicine, UNITED STATES

## Introduction

Maternal antibodies (MatAbs) passively transferred across the placenta and into breast milk are critical for protection against infectious disease and immune development during the first year of life [1]. Passive transfer in the placenta and mammary gland (MG) is dependent on MatAbs binding to crystallizable fragment (Fc) receptors (FcRs) on polarized epithelial cells. For example, immunoglobulin G (IgG) transfers through the placenta by Fc domain binding to the Fc receptor neonatal (FcRn) on syncytiotrophoblasts [2], providing the fetus with a systemic source of protective IgG antibodies [3]. Additionally, maternal dimeric immunoglobulin A (dIgA) antibodies transfer into breast milk by binding to the polymeric immunoglobulin receptor (pIgR) on MG epithelial cells through the antibody joining chain (J-chain) [4] and provide immune protection in the gut while shaping microbiota colonization [4,5]. Yet MatAbs can interfere with the neonatal immune response, particularly after vaccination [6]. This Pearl explores the role of monomeric IgG, the only antibody isotype to cross the placenta, and polymeric IgA, the major antibody species in breast milk, and their Fc domain characteristics on passive transfer to and functional activity in the newborn.

## The IgG Fc domain and its effector functions in the context of MatAb passive transfer

**Fig 1:**
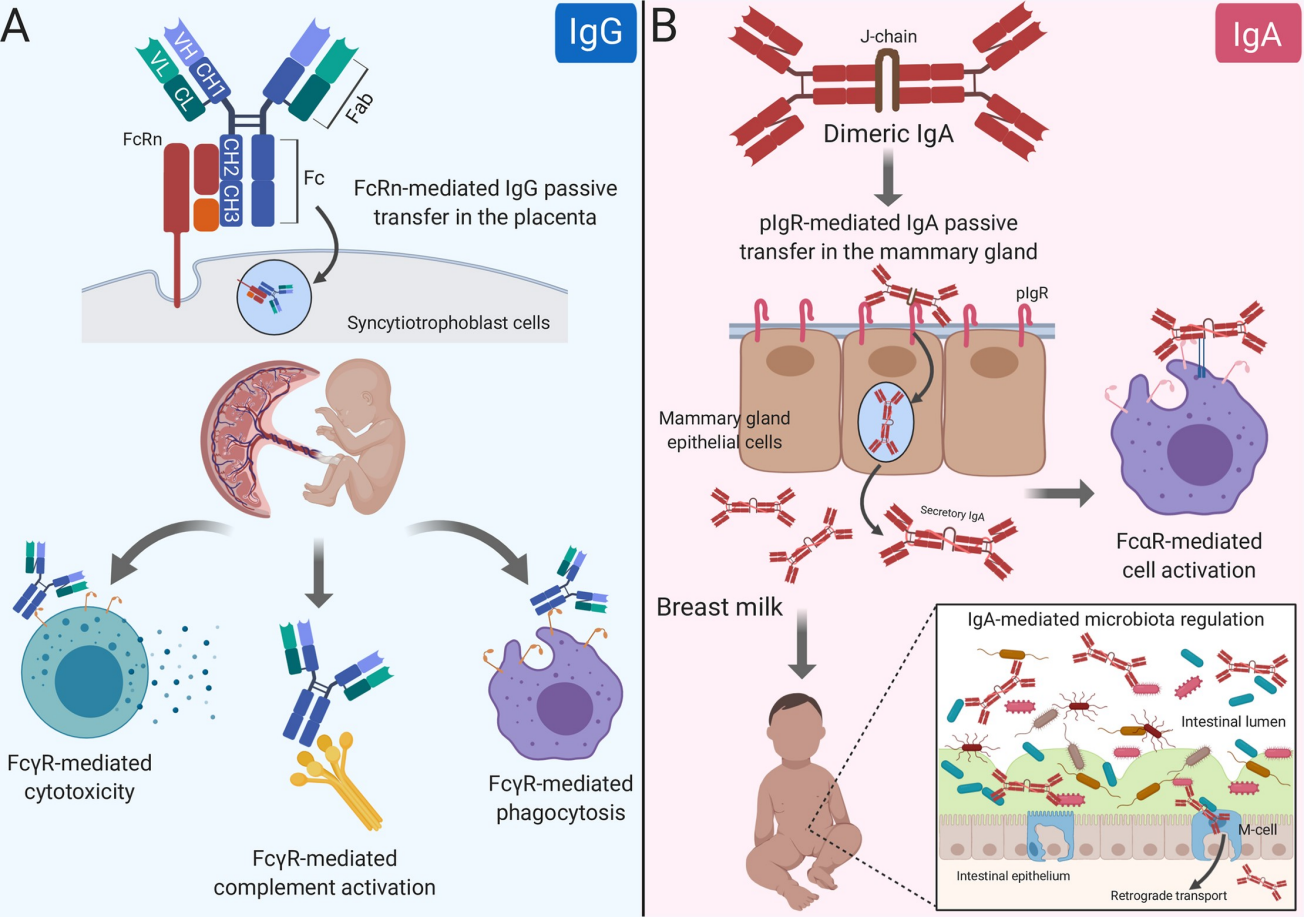
Maternal antibody passive transfer and functional activity in the neonate. (A) IgG passive transfer in the placenta influences FcγR-mediated cell cytotoxicity, phagocytosis, and complement activation in the developing fetus/newborn. (B) IgA passive transfer in the mammary gland results in FcαR- and IgA-mediated cell activation and microbiota regulation, respectively. Fab, antigen-binding fragment; Fc, crystallizable fragment; FcαR, Fc alpha receptor; FcRn, Fc receptor neonatal; FcγR, Fc gamma receptor; IgA, immunoglobulin A; IgG, immunoglobulin G; J-chain, joining chain; pIgR, polymeric immunoglobulin receptor.

Antibodies contain 2 domains that exert a wide range of effector functions. The antigen-binding fragment (Fab) domain binds foreign antigens and drives antibody diversity [7], whereas the Fc is responsible for initiating innate immune cell activation and passive antibody transfer [8]. The classical FcRn-driven IgG transport mechanism is responsible for shuttling IgG within acidified endosomes across the syncytiotrophoblast cell barrier from maternal to fetal circulation (Fig 1A) [2]. Once in the neonate, the IgG Fc domain can engage classical type I Fc gamma (Fcγ) receptors (activating [FcγRI, FcγRIIa, FcγRIIc, FcγRIIIa, FcγRIIIb]; inhibitory [FcγRIIb]) or complement to mediate nonneutralizing functions like antibody-dependent cell-mediated cytotoxicity (ADCC) and antibody-dependent cellular phagocytosis (ADCP), or complement-dependent cytotoxicity (CDC), respectively (Fig 1A) [9]. Nonclassical type II FcRs are C-type lectin receptors, including CD209 (DC-SIGN) and CD23, which bind IgG to facilitate immune complex formation [9]. Considering each family of Fc receptors initiates distinct effector functions, the diversity of the Fc domain allows tailoring of nonneutralizing Fc-mediated activity to protect against viruses like HIV, influenza, and cytomegalovirus [10–12]. Alternatively, pathogens such as dengue virus utilize complement and FcR pathways for antibody-dependent enhancement of disease [13].

**Fig 2:**
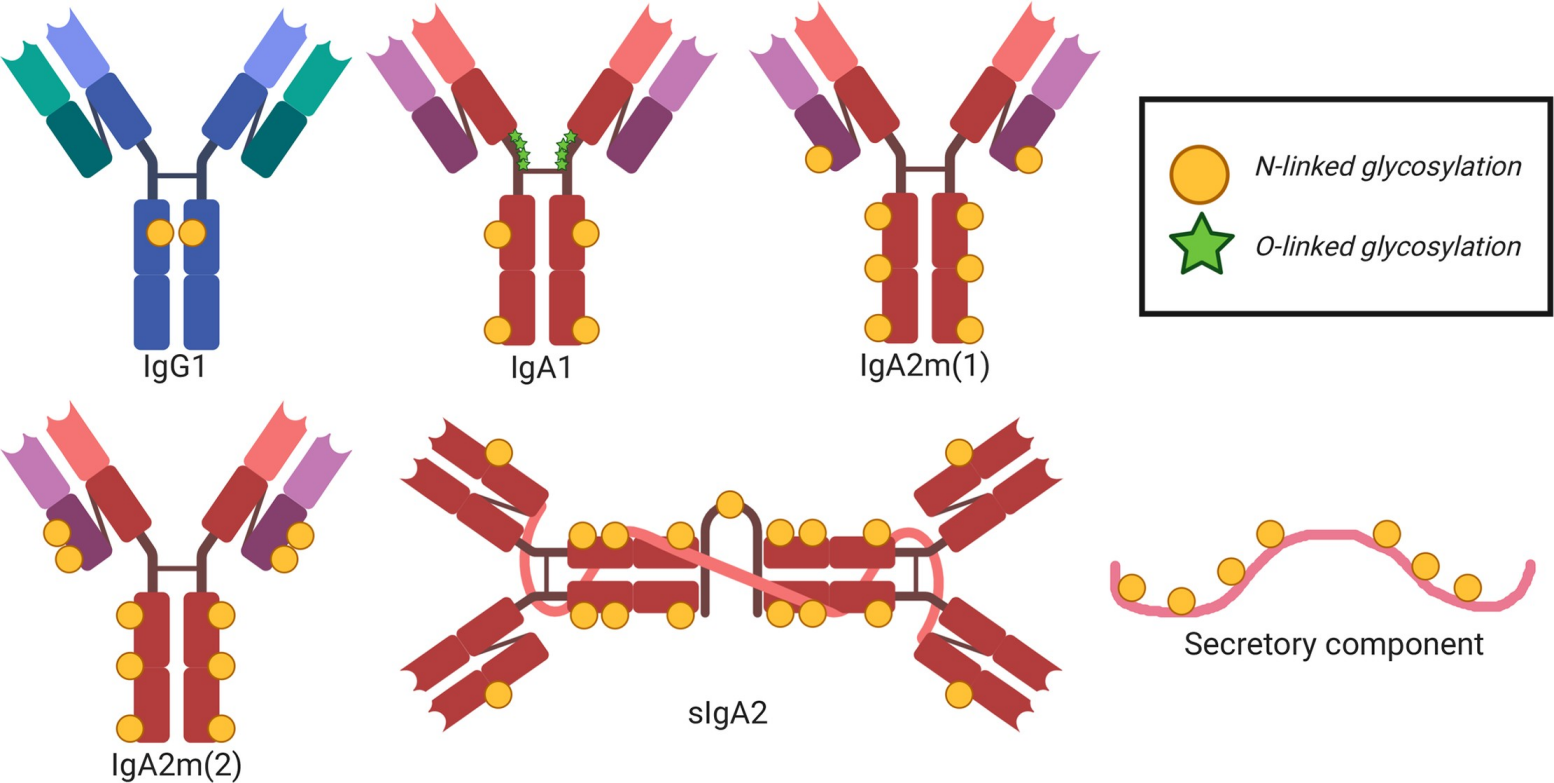
Schematic representation of IgA and IgG glycosylation. N-linked glycosylation is depicted as yellow circles, whereas O-linked glycosylation is depicted as green stars. IgA, immunoglobulin A; IgG, immunoglobulin G; sIgA2, secretory IgA.

The IgG Fc domain mediates considerable heterogeneity of its effector functions depending on the subclass and glycan profile. For example, each IgG subclass (IgG1-4) has one N-glycosylation site in each CH2 domain, an important binding site for FcγRs (Fig 2). Interestingly, there are up to 36 possible antibody glycan profiles that could theoretically be present on each CH2 domain. This allows for combinatorial diversity of the Fc region with 144 different potential functional states for the 4 IgG subclasses [14]. This is relevant in the context of maternal–fetal immunity, as FcRn has different binding affinities to each IgG subclass, which may reflect their placental transfer efficiency [15]. Additionally, recent data suggest that Fc glycan profiles create antibody transfer hierarchies in the placenta of both healthy and HIV-infected pregnant women. For example, in healthy pregnant women, there is a shift toward IgG galactosylated antibodies, which have higher FcRn-binding affinity, are more efficiently transferred across the placenta, and enhance natural killer (NK) cell degranulation and chemokine secretion [16]. Additionally, binding of tetanus toxoid–specific IgG to placental FcγRIIa H131, FcγRIIa R131, and FcγRIIIa F158 (but not canonical FcRn) was positively associated with placental IgG transfer efficiency in HIV-infected women, suggesting that noncanonical placental FcRs may also play a role in IgG placental transfer [17,18]. Fc-mediated differential selection of IgG antibodies in the placenta is likely an adaptive evolutionary mechanism to passively transfer the most effective antibodies to the infant, which can be altered by disease status.

### Do IgA Fc region characteristics influence IgA passive transfer or effector function in breast milk?

IgA antibodies bind their own unique Fc receptors that facilitate epithelial cell transcytosis and innate immune cell activation. dIgA antibodies are composed of 2 monomers, linked by a 15-kDa J-chain. Transport of dIgA into breast milk is dependent on C-terminal binding of the J-chain to a portion of pIgR, known as the secretory component, on the basolateral surface of MG epithelial cells [19]. Without the J-chain, IgA antibodies are secreted as monomers and are not actively transported across the mucosal epithelium [20]. After transport of the J-chain/pIgR complex to the apical portion of the cell, pIgR is cleaved, releasing secretory IgA (sIgA) into breast milk and other mucosal fluids (Fig 1B) [21]. IgA also binds to Fc alpha receptor (FcαR) [22] on the surface of myeloid cells. Monomeric serum IgA induces inhibitory signals, whereas IgA immune complexes have increased avidity to and cross-link FcαRI, resulting in proinflammatory responses [23]. The dominant immunoglobulin class in breast milk sIgA has decreased affinity for FcαRI likely due to steric hindrance from the attached secretory component [24]. Although the opsonic activity of sIgA is poor compared with monomeric and dIgA [25], sIgA can initiate macrophage phagocytosis and neutrophil respiratory burst [26,27]. Further defining the anti- and proinflammatory effects of IgA subclass–FcR interactions would allow fine tuning of breast milk immunity and may represent an attractive therapeutic strategy.

Considering breast milk sIgA protects from pathogenic insult and facilitates maturation of the microbiota in early life [28], understanding breast milk antibody Fc–mediated effector functions is integral to neonatal intestinal health (Fig 1B). This is further supported by the fact that bacteria have evolved mechanisms to block IgA and FcαR interactions [29]. Additionally, it is likely that the more complex and extensive glycosylation pattern of IgA antibodies (Fig 2) impacts effector function in milk. Indeed, mucosal secretions, including breast milk, contain mostly IgA2 [30], which has 2 [IgA2m(1)] or 3 [IgA2m(2)] additional conserved N-glycans compared with IgA1 [31], which dominates in serum [32]. Recent evidence demonstrates that IgA glycan–bacteria interactions regulate gut microbiota composition and metabolism as well as retrograde transport of sIgA immune complexes back to the lamina propria independent of antibody–epitope interactions [33–35]. Additionally, the sialic acid in IgA antibody’s C-terminal tail competes with receptor binding to some viruses, providing an innate line of defense against infection [36]. This is relevant to breast milk IgA passive transfer, considering that the leading causes of severe pediatric gastroenteritis worldwide (rotavirus and norovirus) both utilize sialic acid receptors for intestinal infection [37,38]. Studies are needed to define the mechanisms of Fc-mediated IgA effector functions in breast milk, including their interactions with the developing infant microbiome and protection against intestinal viral infections.

### MatAb interference is influenced by MatAb Fc domain–receptor interactions

Despite the well-documented benefits of MatAbs on early life immunity [3], a mounting body of evidence indicates that MatAbs can inhibit immune responses to certain infant vaccinations [6,39]. A recent meta-analysis demonstrated that MatAbs acquired transplacentally inhibited antibody responses to priming vaccinations and these effects were not overcome by administration of a booster dose [40]. This highlights the “window of susceptibility” that exists for infants when MatAbs are not high enough for seroprotection yet still interfere with infant vaccine responses.

Multiple mechanisms have been proposed to describe both Fab- and Fc-mediated MatAb interference. These include live virus vaccine neutralization, inhibition of B-cell responses by epitope masking [39], and IgG Fc binding to FcγRIIB [41]. Kim and colleagues demonstrated that B-cell responses to a live attenuated measles vaccine were inhibited by passively transferred measles-specific IgG antibodies in a FcγRIIB-dependent manner, suggesting that IgG Fc region characteristics contribute to suppression of the immune response [41]. Additionally, removing the glycans from IgG2b abolished its immunosuppressive activity both in vitro and in vivo [42,43]. Considering that the mechanisms of MatAb interference likely differ depending on vaccine type (live attenuated, inactivated, subunit), route of delivery (oral, subcutaneous [SQ], intramuscular [IM]), and adjuvant formulation, defining glycan-dependent passive transfer of maternal IgG antibody subclasses in the placenta is crucial for developing effective maternal immunization strategies. Although less studied, IgA antibodies in breast milk may also contribute to interference of immune responses to oral infant vaccines such as rotavirus and poliovirus [44,45]. Indeed, the 2 oral rotavirus vaccines Rotarix and Rotateq demonstrate lower efficacy and immunogenicity in infants from some low- and middle-income countries (LMICs) [46,47] where women tend to have higher titers of antirotavirus IgA antibodies and neutralizing activity in milk [48,49]. The high rotavirus neutralizing activity in breast milk of women from LMICs may partially explain the decrease in rotavirus vaccine efficacy; however, IgA MatAb interference is not well defined [49].

Although infant CD4^+^ T-cell responses are mostly unaffected by MatAb interference [6,39], recent work demonstrated that MatAbs dampen mucosal T-cell responses against commensal bacteria [50] and limit the expansion of T follicular helper (T_FH_) cells in the germinal center (GC) [39]. The premature decline in GC T_FH_ cells resulted in the reduction or prevention of plasma cell and memory B-cell generation in a MatAb- and antigen dose–dependent manner [39]. Interestingly, at low or intermediate titers, MatAbs did not prevent the induction of memory B cells, suggesting a gradient effect of MatAbs on infant immune responses [39]. Defining the functional consequences of MatAb gradients will be essential for infant vaccine design and immunization timing. For example, and in addition to previously discussed mechanisms, there is evidence that preexisting antibodies can promote higher affinity antibody responses due to competitive binding in the GC [51] and increased uptake and antigen presentation through immune complexes (ICs) [52] in a Fc glycosylation–dependent manner [53,54]. However, more research is needed to determine the effects of these mechanisms in the setting of passively transferred MatAbs and infant GC responses.

### Harnessing MatAb Fc region characteristics and receptor interactions to fine-tune maternal immunizations that maximize infant protection

Passive transfer of MatAbs is central to pathogen protection and immune system development in early life. However, MatAb-mediated interference dampens antibody responses to vaccinations, leaving children more susceptible to infections while increasing transmission rates to unvaccinated cohorts. Recent work demonstrated that maternal IgG antibodies are differentially transferred across the placenta in an Fc glycan–dependent manner [16,17]. Considering vaccination strategies could direct antigen-specific antibody glycosylation [55], defining MatAb glycan profiles represents an adaptable and powerful mechanism to fine-tune maternal immunizations that maximize infant protection while limiting MatAb interference. Future studies are needed to determine (1) how maternal vaccination and their distinct adjuvant mixtures alter IgG Fc domain glycosylation and whether certain glycan profiles are associated with IgG Fc-mediated MatAb interference and (2) whether or not the IgA Fc domain regulates passive transfer in the MG or effector function in breast milk. Defining the molecular mechanisms of Fc-mediated functional activity at the maternal–fetal/neonatal interface is critical for developing next-generation maternal vaccines and antibody-based therapeutics to improve the health of the mother–neonatal dyad.
